# Discontinuation of alendronate and administration of bone-forming agents after surgical nailing may promote union of atypical femoral fractures in patients on long-term alendronate therapy

**DOI:** 10.1186/1756-0500-6-11

**Published:** 2013-01-11

**Authors:** Tsung-Li Lin, Shyu-Jye Wang, Yi-Chin Fong, Chin-Jung Hsu, Horng-Chaung Hsu, Chun-Hao Tsai

**Affiliations:** 1 Department of Orthopaedic Surgery, China Medical University Hospital, School of Medicine, China Medical University, Taichung, Taiwan

**Keywords:** Atypical femoral fracture, Union, Alendronate, Bone-forming agents

## Abstract

**Background:**

Long-term alendronate therapy may lead to atypical femoral fractures in a very few patients. However, the management protocol to optimize fracture healing remains undetermined. The purpose of this study was to describe the time to union of atypical femoral fractures after surgical nailing in patients on long-term alendronate therapy, with continuation of alendronate or discontinuation of alendronate, and administration of bone-forming agents.

**Findings:**

From January 2004 to December 2011, the records of patients at our institution on long-term alendronate therapy for more than 36 months and sustaining atypical femoral fractures that had undergone surgical nailing, with continuation of alendronate or discontinuation of alendronate and administration of bone-forming agents were reviewed.

During the 8-year study period, we treated 10 atypical femoral fractures with surgical nailing in 7 consecutive osteopenic or osteoporotic patients on long-term alendronate therapy for more than 36 months. There were no post-operative complications or needs for revision surgery. All fractures achieved union during follow-up. Mean union time was 23.5 months (range, 18–31 months) for 6 fractures with continuation of alendronate after surgery, and 4.5 months (range, 4–5 months) for 4 fractures with discontinuation of alendronate and administration of bone-forming agents after surgery.

**Conclusions:**

Discontinuation of alendronate and administration of bone-forming agents after surgical nailing may promote union of atypical femoral fractures in patients on long-term alendronate therapy.

## Findings

### Background

Postmenopausal osteoporosis is commonly treated with alendronate, one of the bisphosphonates used for the prevention and treatment of osteoporotic fractures. However, long-term alendronate therapy may lead to atypical femoral fractures in a very few patients, but the management protocol to optimize fracture healing remains undetermined.

Prior studies reported delayed healing of atypical femoral fractures with continuation of alendronate [1-3]. There is evidence that bone-forming agents, such as strontium ranelate or teriparatide, may improve bone turnover and microarchitecture [4-6]. There have been reports that indicate bone-forming agents exert a rapid bone anabolic effect on unhealed atypical fractures associated with chronic bisophosphonate use [2,3,7].

Herein, we describe the use of bone-forming agents in the successful management of atypical femoral fractures after surgical nailing in patients on long-term alendronate therapy.

## Methods

The records of patients who sustained subtrochanteric or diaphyseal femoral fractures and received surgery between January 2004 and December 2011 at our institution were reviewed. The definition of an atypical femoral fracture is based on the ASBMR (American Society for Bone and Mineral Research) criteria. That is, fractures were located in subtrochanteric or diaphyseal of femur, typically radiographic features of simple transverse or short oblique configuration without comminution were present, complete fractures extend through both cortices and may be associated with a medial spike, incomplete fractures involved only the lateral cortex, and with minimal or no trauma mechanism [8]. The inclusions criteria were that patients received oral alendronate 70 mg once weekly more than 36 months, and the fractures received surgery on the day of injury or the next day. Patients with histories of malignancy, pathologic fractures, metabolic bone diseases, receiving glucocorticoid or other antiresorptives, were excluded.

Diaphyseal femoral fractures were fixed with antegrade intramedullary nailing (Howmedica, Rutherford, NJ, U.S.A.) and subtrochanteric femoral fractures were fixed with cephalomedullary nailing (PFNA II, Synthes, Oberdorf, Switzerland). All fractures were fixed with a closed method on a fracture table.

Follow-up visits and radiographs were performed in our outpatient clinics at 4, 8, 12, and 16 weeks and variably thereafter postoperatively. Bony union was defined as bridging of 3 of the 4 cortices on anteroposterior and lateral radiographs.

## Results

During the 8-year study period, there were 150 subtrochanteric and 1104 diaphyseal femoral fractures treated with surgery at our institution. There were 10 atypical femoral fractures in 7 consecutive osteopenic or osteoporotic patients on alendronate therapy for more than 36 months. All patients were female with a mean age of 80.5 years (range, 74–88 years). The mean duration of alendronate therapy was 66.1 months (range, 36–112 months). All were complete atypical fractures due to an atraumatic mechanism, such as slipping down or simple sprain injury. The femoral fracture was subtrochanteric in 1 and diaphyseal in 9. Three patients had sequential femoral diaphyseal fractures (Table 1). The minimum follow-up time was 12 months, and the mean follow-up was 18.9 months (range, 12–31 months).

**Table 1:** Patient demographics, postoperative treatment, and union time

**Case**	**Age (y)**	**Sex**	**Side**	**Fracture mechanism**	**Fracture location**	**Duration of ALN therapy (m)**	**BMD (T-score)**	**Surgery**	**Postoperative treatment**	**Union time (m)**
1	84	F	R	Sprain	FS	36	−2.4	IMN	ALN	24
			L*	Sprain	FS	62	NA	IMN	ALN	31
2	88	F	R	Slipping fall	FS	94	−2.8	IMN	ALN	20
3	80	F	R	Sprain	FS	65	−4.2	IMN	ALN	18
			L*	Sprain	FS	112	NA	IMN	ALN	18
4	83	F	R	Slipping fall	FS	50	−1.0	IMN	ALN	30
5	77	F	L	Sprain	FS	63	−3.0	IMN	TPTD	5
			R**	Sprain	FS	63	NA	IMN	SR	4
6	78	F	R	Slipping fall	FS	52	−1.8	IMN	SR	4
7	74	F	R	Slipping fall	ST	64	−1.7	CMN	SR	5

ALN, alendronate; BMD, bone mineral density; FS, femoral shaft; ST, subtrochanteric; NA, not available; IMN, intramedullary nail; CMN, cephalomedullary nail; TPTD, teriparatide; SR, strontium ranelate.

*Sequential contralateral fractures occurred in the patient continued alendronate after surgery.

**Discontinued bone-forming agent after union of the initial fracture, and sequential contralateral femoral fracture noted 24 months later.

In 6 fractures treated earlier in the study period, alendronate was continued after surgery. In 4 fractures treated later in the study period, alendronate was discontinued and bone-forming agents were administrated immediately after surgery. The bone-forming agents were 2 g of strontium ranelate orally daily or 20 μg of teriparatide subcutaneously daily. All fractures achieved union during follow-up. Mean union time was 23.5 months (range, 18–31 months) for 6 fractures with continuation of alendronate after surgery, and 4.5 months (range, 4–5 months) for 4 fractures with discontinuation of alendronate and administration of bone-forming agents after surgery.

**Figure 1:**
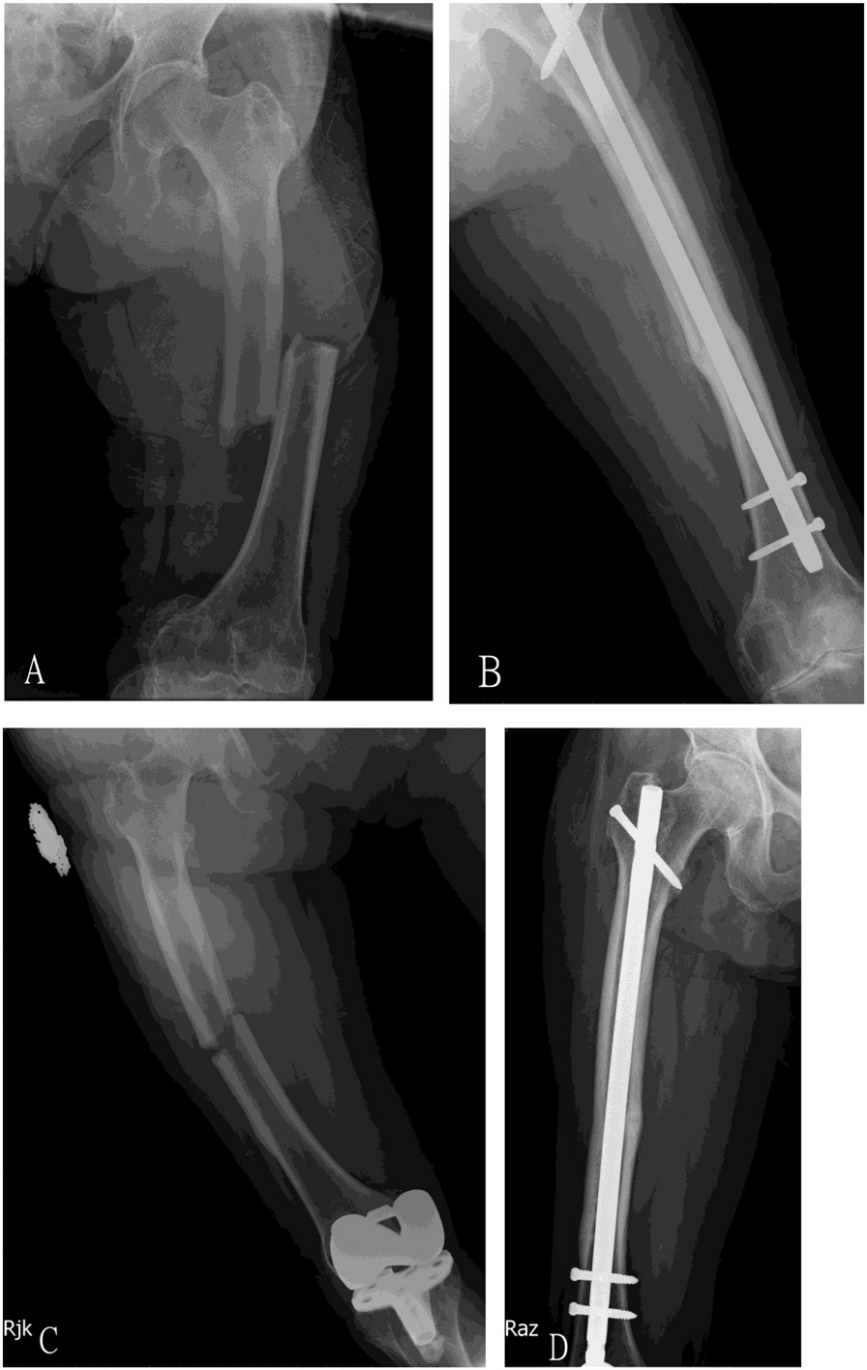
**A 77 year-old female suffered from sequential femoral diaphyseal fractures.** (**A**) The initial femoral fracture happened after 63 months of alendronate therapy. (**B**) The union was achieved at 5 months by discontinuation of alendronate and administration of bone-forming agent after surgery. (**C**) She then received a total knee arthroplasty for right knee osteoarthritis 11 months later. However, contralateral femoral fracture was noted 24 months after the initial fracture. (**D**) After administration of bone-forming agent, bone was healed on 4^th^ month post-operatively.

One case (case No. 5) of sequential femoral diaphyseal fractures revealed an interesting finding. The initial fracture occurred after 63 months of alendronate therapy, and the union was achieved at 5 months by discontinuation of alendronate and administration of bone-forming agent after surgery (Figure 1A and 1B). Then bone-forming agent was stopped after union of fracture. The patient then received a total knee arthroplasty for right knee osteoarthritis 11 months later. However, a contralateral atypical femoral fracture was noted 24 months after the initial fracture. The patient did not use alendronate during the 24 months period. After administration of bone-forming agent, bone was healed on 4^th^ month post-operatively (Figure 1C and 1D).

## Discussion

In the retrospective review of the time to union of atypical femoral fractures after surgical nailing in patients on alendronate therapy for more than 36 months, we found that discontinuation of alendronate and administration of bone-forming agents significantly shortened the time to union as compared to continuation of alendronate. We performed surgical nailing in all fractures through a closed method. Although delayed union was noted in follow-up of all fractures, we chose observation and waiting rather than performing revision surgery because 6 fractures with continuation of alendronate after surgery showed continuing callus formation as compared with previous radiographs during follow up. In addition, there were neither postoperative complications nor revision surgeries. However, Weil et al. described healing of 17 atypical femoral fractures after intramedullary nailing in only 10 (54%) patients, with 7 (46%) patients requiring revision surgery. These secondary procedures included nail dynamization in 4 cases, exchange nailing in 2, and plating in 1. They supposed the higher failure rate seen with bisphosphonate-related fractures might have been due to an impaired bone healing process rather than differences in surgical technique, and qualitative bone defects caused by prolonged bisphosphonate therapy. The authors suggested more aggressive intraoperative biologic augmentation, such as primary bone grafting, the use of human recombinant bone morphogenic protein, or treatment with systemic parathyroid hormone analogs due to apparently impaired bone metabolism associated with bisphosphonate therapy [9].

Atypical femoral fractures are stress fractures associated with suppression of bone remodeling induced by bisphosphonates [10]. Delayed union in such fractures was reported in previous studies [1-3,8,11]. Some studies have suggested potential negative effects of bisphosphonate on the fracture healing process, leading to the suggestion that treatment should be discontinued during the healing phase [1,8,12,13]. Some clinical evidence and a large body of animal data indicate bone-forming agents, such as strontium ranelate or teriparatide, may accelerate fracture healing [14-18]. An increase in biomarkers was also observed in patients with severe osteoporosis who were undergoing treatment with bone-forming agents, despite previous treatment with bisphosphonates [19]. Bone-forming agents have been shown to expert a rapid bone anabolic effect on unhealed atypical fractures associated with chronic alendronate use [2,3,7]. In this study, the time to union of fractures with continuation of alendronate was longer than fractures with discontinuation of alendronate and administration of bone-forming agents after surgery. However, our finding is from regular dosage of bone-forming agents for antiosteoporosis. There is possibility that increasing the dosage of bone-forming agents could also reduce the months and increase the union at a faster space and this provides a platform to evaluate further.

We have no control group, that is, lack of fracture, which discontinued alendronate and did not received bone-forming agents after surgery. Even though previous studies have reported that bone-forming agents healed atypical fractures and even with the time course of fracture healing in our patient, we are still not certain that bone-forming agents played a primary role in the positive response to therapy or alendronate discontinuation may have played secondary roles. The bone-forming agent effects might be reduced if alendronate is discontinued, for although bone repair requires increased bone formation, remodeling is also necessary for bone repair. Odvina et al. reported delayed union with mean time 9.2 months (range 8 to 12) of 4 atypical femoral fractures despite discontinuing alendronate therapy [1]. Visekruna et al. also reported no fracture healing after 12, 22 and 6 months after alendronate stopped in 3 atypical femoral fractures. However, Visekruna speculated that delayed union might be attributable to the fact that the patients were taking estrogen or other medications (glucocorticoids, hormone replacement therapy, or raloxifene) that might have resulted in further suppression of bone turnover [2]. Capeci et al. treated 7 patients who sustained bilateral simultaneous or sequential fractures with femoral nailing and discontinuation of alendronate and reported uneventful healing at an average of 4 months (range, 3 to 5 months) [20]. Alendronate therapy was discontinued at the time of the second fracture fixation in the patients with sequential fractures and at the time of injury in the patient with simultaneous fractures. The 7 patients were not taking glucocorticoids, hormone replacement therapy, or raloxifene. The normal time to fracture union in Capeci’s study is in contrast to that noted in most reports, which have shown delayed healing in these patients. However, 4 of these fractures were impending subtrochanteric stress fractures, which received prophylactic fixation and the usage of bone-forming agents or not was not documented. The time to union of fractures which the alendronate has been discontinued and no bone-forming agents have been applied seems longer than fractures with discontinuation of alendronate and administration of bone-forming agents after surgery.

The sequential femoral diaphyseal fractures revealed an interesting finding. We believed bone turnover may remain suppressed several months or years after discontinuation of alendronate. A long skeletal half-life of alendronate which impairs fracture healing has been described [21], but there was no evidence indicating what duration of treatment with bone-forming agents would counteract the effect of alendronate, or that bone-forming agents could prevent sequential fracture. Further study is needed for this issue.

The major limitations of this study are those inherent within a retrospective case series. This was a small number of patients with limited data available, and statistical comparison was therefore not possible. In addition, not all patients were evaluated for biomarkers as well for bone pathology.

## Conclusions

Discontinuing of alendronate and administration of bone-forming agents after surgical nailing may promote union of atypical femoral fractures in patients on long-term alendronate therapy. Further studies are needed to establish optimal therapeutic guidelines.

### Availability of supporting data

The data sets supporting the findings are included within the article.

## Abbreviations

(ASBMR): American society for bone and mineral research.

## Competing interests

The authors declare that they have no competing interests.

## Authors’ contributions

TLL and CHT designed the research and wrote the paper; SJW and CJH conducted the research; YCF and HCH analyzed the data; CHT had primary responsibility for final content. All authors read and approved the final manuscript.
